# RustHorn: CHC-Based Verification for Rust Programs

**DOI:** 10.1007/978-3-030-44914-8_18

**Published:** 2020-04-18

**Authors:** Yusuke Matsushita, Takeshi Tsukada, Naoki Kobayashi

**Affiliations:** ETH Zurich, Zurich, Switzerland; The University of Tokyo, Tokyo, Japan

## Abstract

Reduction to the satisfiablility problem for constrained Horn clauses (CHCs) is a widely studied approach to automated program verification. The current CHC-based methods for pointer-manipulating programs, however, are not very scalable. This paper proposes a novel translation of pointer-manipulating Rust programs into CHCs, which clears away pointers and heaps by leveraging ownership. We formalize the translation for a simplified core of Rust and prove its correctness. We have implemented a prototype verifier for a subset of Rust and confirmed the effectiveness of our method.

### 1 Introduction

Reduction to *constrained Horn clauses (CHCs)* is a widely studied approach to automated program verification [22,6]. A CHC is a Horn clause [30] equipped with constraints, namely a formula of the form $\varphi \Longleftarrow \psi_0 \wedge \cdots \wedge \psi_{k-1}$, where $\varphi$ and $\psi_0, \ldots, \psi_{k-1}$ are either an atomic formula of the form $f(t_0, \ldots, t_{n-1})$ ($f$ is a *predicate variable* and $t_0, \ldots, t_{n-1}$ are terms), or a constraint (e.g. $a < b+1$).[1] We call a finite set of CHCs a *CHC system* or sometimes just CHC. *CHC solving* is an act of deciding whether a given CHC system $S$ has a *model*, i.e. a valuation for predicate variables that makes all the CHCs in $S$ valid. A variety of program verification problems can be naturally reduced to CHC solving.

For example, let us consider the following C code that defines McCarthy's 91 function.

```
int mc91(int n) {
  if (n > 100) return n - 10; else return mc91(mc91(n + 11));
}
```

Suppose that we wish to prove `mc91`$(n)$ returns 91 whenever $n \leq 101$ (if it terminates). The wished property is equivalent to the satisfiability of the following CHCs, where $Mc91(n,r)$ means that `mc91`$(n)$ returns $r$ if it terminates.

$$Mc91(n,r) \Longleftarrow n > 100 \wedge r = n - 10$$

⋆ The full version of this paper is available as [47].

[1] Free variables are universally quantified. Terms and variables are governed under sorts (e.g. `int`, `bool`), which are made explicit in the formalization of § 3.

$$Mc91(n,r) \impliedby n \le 100 \land Mc91(n+11, res') \land Mc91(res', r)$$
$$r = 91 \impliedby n \le 101 \land Mc91(n,r)$$

The property can be verified because this CHC system has a model:

$$Mc91(n,r) \;:\Longleftrightarrow\; r = 91 \lor (n > 100 \land r = n - 10).$$

A CHC solver provides a common infrastructure for a variety of programming languages and properties to be verified. There have been effective CHC solvers [40,18,29,12] that can solve instances obtained from actual programs[2] and many program verification tools [23,37,25,28,38,60] use a CHC solver as a backend.

However, the current CHC-based methods do not scale very well for programs using *pointers*, as we see in § 1.1. We propose a novel method to tackle this problem for pointer-manipulating programs under *Rust-style ownership*, as we explain in § 1.2.

#### 1.1 Challenges in Verifying Pointer-Manipulating Programs

The standard CHC-based approach [23] for pointer-manipulating programs represents the memory state as an *array*, which is passed around as an argument of each predicate (cf. the *store-passing style*), and a pointer as an index.

For example, a pointer-manipulating variation of the previous program

```
void mc91p(int n, int* r) {
  if (n > 100) *r = n - 10;
  else { int s; mc91p(n + 11, &s); mc91p(s, r); }
}
```

is translated into the following CHCs by the array-based approach:[3]

$$Mc91p(n,r,h,h') \impliedby n > 100 \land h' = h\{r \leftarrow n - 10\}$$
$$Mc91p(n,r,h,h') \impliedby n \le 100 \land Mc91p(n+11, s, h, h'') \land Mc91p(h''[s], r, h'', h')$$
$$h'[r] = 91 \impliedby n \le 101 \land Mc91p(n,r,h,h').$$

*Mc91p* additionally takes two arrays $h, h'$ representing the (heap) memory states before/after the call of `mc91p`. The second argument $r$ of *Mc91p*, which corresponds to the pointer argument `r` in the original program, is an index for the arrays. Hence, the assignment `*r = n - 10` is modeled in the first CHC as an update of the $r$-th element of the array. This CHC system has a model

$$Mc91p(n,r,h,h') \;:\Longleftrightarrow\; h'[r] = 91 \lor (n > 100 \land h'[r] = n - 10),$$

which can be found by some array-supporting CHC solvers including Spacer [40], thanks to evolving SMT-solving techniques for arrays [62,10].

However, the array-based approach has some shortcomings. Let us consider, for example, the following innocent-looking code.[4]

[2] For example, the above CHC system on *Mc91* can be solved instantly by many CHC solvers including Spacer [40] and HoIce [12].

[3] $h\{r \leftarrow v\}$ is the array made from $h$ by replacing the value at index $r$ with $v$. $h[r]$ is the value of array $h$ at index $r$.

[4] `rand()` is a non-deterministic function that can return any integer value.

```
bool just_rec(int* ma) {
  if (rand() >= 0) return true;
  int old_a = *ma; int b = rand(); just_rec(&b);
  return (old_a == *ma);
}
```

It can immediately return `true`; or it recursively calls itself and checks if the target of `ma` remains unchanged through the recursive call. In effect this function *does nothing* on the allocated memory blocks, although it can possibly modify some of the unused parts of the memory.

Suppose we wish to verify that `just_rec` never returns `false`. The standard CHC-based verifier for C, SeaHorn [23], generates a CHC system like below:[5][6]

$$\begin{aligned}
&\mathit{JustRec}(ma, h, h', r) \impliedby h' = h \land r = \mathsf{true} \\
&\mathit{JustRec}(ma, h, h', r) \impliedby mb \neq ma \land h'' = h\{mb \leftarrow b\} \\
&\qquad\qquad \land \mathit{JustRec}(mb, h'', h', r') \land r = (h[ma] == h'[ma]) \\
&r = \mathsf{true} \impliedby \mathit{JustRec}(ma, h, h', r)
\end{aligned}$$

Unfortunately the CHC system above is *not* satisfiable and thus SeaHorn issues a false alarm. This is because, in this formulation, $mb$ may not necessarily be completely fresh; it is assumed to be different from the argument $ma$ of the current call, but may coincide with $ma$ of some deep ancestor calls.[7]

The simplest remedy would be to explicitly specify the way of memory allocation. For example, one can represent the memory state as a pair of an array $h$ and an index $sp$ indicating the maximum index that has been allocated so far.

$$\begin{aligned}
&\mathit{JustRec}_+(ma, h, sp, h', sp', r) \impliedby h' = h \land sp' = sp \land r = \mathsf{true} \\
&\mathit{JustRec}_+(ma, h, sp, h', sp', r) \impliedby mb = sp'' = sp + 1 \land h'' = h\{mb \leftarrow b\} \\
&\qquad\qquad \mathit{JustRec}_+(mb, h'', sp'', h', sp', r') \land r = (h[ma] == h'[ma]) \\
&r = \mathsf{true} \impliedby \mathit{JustRec}_+(ma, h, sp, h', sp', r) \land ma \leq sp
\end{aligned}$$

The resulting CHC system now has a model, but it involves quantifiers:

$$\mathit{JustRec}_+(ma, h, sp, h', sp', r) \;:\Longleftrightarrow\; r = \mathsf{true} \land \forall\, i \leq sp.\, h[i] = h'[i]$$

Finding quantified invariants is known to be difficult in general despite active studies on it [41,2,36,26,19] and most current array-supporting CHC solvers give up finding quantified invariants. In general, much more complex operations on pointers can naturally take place, which makes the universally quantified invariants highly involved and hard to automatically find. To avoid complexity of models, CHC-based verification tools [23,24,37] tackle pointers by pointer analysis [61,43]. Although it does have some effects, the current applicable scope of pointer analysis is quite limited.

5 ==, !=, >=, && denote binary operations that return boolean values.

6 We omitted the allocation for `old_a` for simplicity.

7 Precisely speaking, SeaHorn tends to even omit shallow address-freshness checks like $mb \neq ma$.

#### 1.2 Our Approach: Leverage Rust's Ownership System

This paper proposes a novel approach to CHC-based verification of pointer-manipulating programs, which makes use of *ownership* information to avoid an explicit representation of the memory.

*Rust-style Ownership.* Various styles of *ownership/permission/capability* have been introduced to control and reason about usage of pointers on programming language design, program analysis and verification [13,31,8,31,9,7,64,63]. In what follows, we focus on the ownership in the style of the Rust programming language [46,55].

Roughly speaking, the ownership system guarantees that, for each memory cell and at each point of program execution, either (i) only one alias has the *update* (write & read) permission to the cell, with any other alias having *no* permission to it, or (ii) some (or no) aliases have the *read* permission to the cell, with no alias having the update permission to it. In summary, *when an alias can read some data* (with an update/read permission), *any other alias cannot modify the data.*

As a running example, let us consider the program below, which follows Rust's ownership discipline (it is written in the C style; the Rust version is presented at Example 1):

```
int* take_max(int* ma, int* mb) {
  if (*ma >= *mb) return ma; else return mb;
}
bool inc_max(int a, int b) {
  {
    int* mc = take_max(&a, &b);  // borrow a and b
    *mc += 1;
  }                              // end of borrow
  return (a != b);
}
```

Figure 1 illustrates which alias has the update permission to the contents of `a` and `b` during the execution of `take_max(5,3)`.

A notable feature is *borrow*. In the running example, when the pointers `&a` and `&b` are taken for `take_max`, the *update permissions* of `a` and `b` are *temporarily transferred* to the pointers. The original variables, `a` and `b`, *lose the ability to access their contents* until the end of borrow. The function `take_max` returns a pointer having the update permission until the end of borrow, which justifies the *update operation* `*mc += 1`. In this example, the end of borrow is at the end of the inner block of `inc_max`. At this point, *the permissions are given back* to the original variables `a` and `b`, allowing to compute `a != b`. Note that `mc` can point to `a` and also to `b` and that this choice is determined *dynamically*. The values of `a` and `b` after the borrow *depend on the behavior of the pointer* `mc`.

The end of each borrow is statically managed by a *lifetime*. See § 2 for a more precise explanation of ownership, borrow and lifetimes.

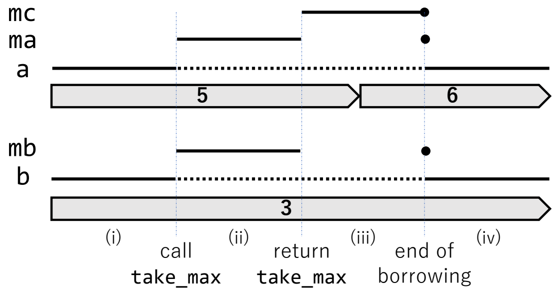

**Fig. 1.** Values and aliases of $a$ and $b$ in evaluating `inc_max(5,3)`. Each line shows each variable's permission timeline: a solid line expresses the update permission and a bullet shows a point when the borrowed permission is given back. For example, `b` has the update permission to its content during (i) and (iv), but not during (ii) and (iii) because the pointer `mb`, created at the call of `take_max`, *borrows* `b` until the end of (iii).

*Key Idea.* The key idea of our method is to *represent a pointer* `ma` *as a pair* $\langle a, a_\circ \rangle$ *of the current target value* $a$ *and the target value* $a_\circ$ *at the end of borrow.*[8][9] This representation employs *access to the future information* (it is related to *prophecy variables*; see § 5). This simple idea turns out to be very powerful.

In our approach, the verification problem "Does `inc_max` always return `true`?" is reduced to the satisfiability of the following CHCs:

$$\begin{aligned}
&\mathit{TakeMax}(\langle a, a_\circ \rangle, \langle b, b_\circ \rangle, r) \iff a \geq b \land b_\circ = b \land r = \langle a, a_\circ \rangle \\
&\mathit{TakeMax}(\langle a, a_\circ \rangle, \langle b, b_\circ \rangle, r) \iff a < b \land a_\circ = a \land r = \langle b, b_\circ \rangle \\
&\mathit{IncMax}(a, b, r) \iff \mathit{TakeMax}(\langle a, a_\circ \rangle, \langle b, b_\circ \rangle, \langle c, c_\circ \rangle) \land c' = c + 1 \\
&\qquad\qquad\qquad \land c_\circ = c' \land r = (a_\circ \mathrel{!=} b_\circ) \\
&r = \mathsf{true} \iff \mathit{IncMax}(a, b, r).
\end{aligned}$$

The mutable reference `ma` is now represented as $\langle a, a_\circ \rangle$, and similarly for `mb` and `mc`. The first CHC models the then-clause of `take_max`: the return value is `ma`, which is expressed as $r = \langle a, a_\circ \rangle$; in contrast, `mb` is released, which *constrains* $b_\circ$, the value of `b` at the end of borrow, to the current value $b$. In the clause on *IncMax*, $mc$ is represented as a pair $\langle c, c_\circ \rangle$. The constraint $c' = c + 1 \land c_\circ = c'$ models the increment of `mc` (in the phase (iii) in Fig. 1). Importantly, the final check `a != b` is simply expressed as $a_\circ \mathrel{!=} b_\circ$; the updated values of `a`/`b` are available as $a_\circ / b_\circ$. Clearly, the CHC system above has a simple model.

Also, the `just_rec` example in § 1.1 can be encoded as a CHC system

$$\begin{aligned}
&\mathit{JustRec}(\langle a, a_\circ \rangle, r) \iff a_\circ = a \land r = \mathsf{true} \\
&\mathit{JustRec}(\langle a, a_\circ \rangle, r) \iff mb = \langle b, b_\circ \rangle \land \mathit{JustRec}(mb, r') \\
&\qquad\qquad\qquad \land a_\circ = a \land r = (a == a_0)
\end{aligned}$$

[8] Precisely, this is the representation of a pointer with a borrowed update permission (i.e. *mutable reference*). Other cases are discussed in § 3.

[9] For example, in the case of Fig. 1, when `take_max` is called, the pointer `ma` is $\langle 5, 6 \rangle$ and `mb` is $\langle 3, 3 \rangle$.

$$r = \mathsf{true} \;\Longleftarrow\; \mathit{JustRec}(\langle a, a_\circ \rangle, r).$$

Now it has a simple model: $\mathit{JustRec}(\langle a, a_\circ \rangle, r) \;:\Longleftrightarrow\; r = \mathsf{true} \land a_\circ = a$. Remarkably, arrays and quantified formulas are not required to express the model, which allows the CHC system to be easily solved by many CHC solvers. More advanced examples are presented in § 3.4, including one with destructive update on a singly-linked list.

*Contributions.* Based on the above idea, we formalize the translation from programs to CHC systems for a core language of Rust, prove correctness (both soundness and completeness) of the translation, and confirm the effectiveness of our approach through preliminary experiments. The core language supports, among others, recursive types. Remarkably, our approach enables us to automatically verify some properties of a program with destructive updates on recursive data types such as lists and trees.

The rest of the paper is structured as follows. In § 2, we provide a formalized core language of Rust supporting recursions, lifetime-based ownership and recursive types. In § 3, we formalize our translation from programs to CHCs and prove its correctness. In § 4, we report on the implementation and the experimental results. In § 5 we discuss related work and in § 6 we conclude the paper.

### 2 Core Language: Calculus of Ownership and Reference

We formalize a core of Rust as *Calculus of Ownership and Reference (COR)*, whose design has been affected by the safe layer of $\lambda_{\mathsf{Rust}}$ in the RustBelt paper [32]. It is a typed procedural language with a Rust-like ownership system.

#### 2.1 Syntax

The following is the syntax of COR.

$$\begin{aligned}
&\text{(program) } \Pi \;::=\; F_0 \cdots F_{n-1} \\
&\text{(function definition) } F \;::=\; \mathsf{fn}\, f\, \Sigma\, \{L_0 \colon S_0 \;\cdots\; L_{n-1} \colon S_{n-1}\} \\
&\text{(function signature) } \Sigma \;::=\; \langle \alpha_0, \dots, \alpha_{m-1} \mid \alpha_{a_0} \le \alpha_{b_0}, \dots, \alpha_{a_{l-1}} \le \alpha_{b_{l-1}} \rangle \\
&\qquad (x_0 \colon T_0, \dots, x_{n-1} \colon T_{n-1}) \to U \\
&\text{(statement) } S \;::=\; I;\, \mathsf{goto}\, L \mid \mathsf{return}\, x \\
&\qquad \mid \mathsf{match}\, {*x}\, \{\mathsf{inj}_0 {*y_0} \to \mathsf{goto}\, L_0,\; \mathsf{inj}_1 {*y_1} \to \mathsf{goto}\, L_1\} \\
&\text{(instruction) } I \;::=\; \mathsf{let}\, y = \mathsf{mutbor}_\alpha\, x \mid \mathsf{drop}\, x \mid \mathsf{immut}\, x \mid \mathsf{swap}({*x}, {*y}) \\
&\qquad \mid \mathsf{let}\, {*y} = x \mid \mathsf{let}\, y = {*x} \mid \mathsf{let}\, {*y} = \mathsf{copy}\, {*x} \mid x \;\mathsf{as}\; T \\
&\qquad \mid \mathsf{let}\, y = f\langle \alpha_0, \dots, \alpha_{m-1} \rangle (x_0, \dots, x_{n-1}) \\
&\qquad \mid \mathsf{intro}\, \alpha \mid \mathsf{now}\, \alpha \mid \alpha \le \beta \\
&\qquad \mid \mathsf{let}\, {*y} = \mathit{const} \mid \mathsf{let}\, {*y} = {*x}\; \mathit{op}\; {*x'} \mid \mathsf{let}\, {*y} = \mathsf{rand}() \\
&\qquad \mid \mathsf{let}\, {*y} = \mathsf{inj}_i^{T_0+T_1}\, {*x} \mid \mathsf{let}\, {*y} = ({*x_0}, {*x_1}) \mid \mathsf{let}\, ({*y_0}, {*y_1}) = {*x} \\
&\text{(type) } T, U \;::=\; X \mid \mu X.T \mid P\,T \mid T_0 + T_1 \mid T_0 \times T_1 \mid \mathsf{int} \mid \mathsf{unit} \\
&\text{(pointer kind) } P \;::=\; \mathsf{own} \mid R_\alpha \quad \text{(reference kind) } R \;::=\; \mathsf{mut} \mid \mathsf{immut}
\end{aligned}$$

$$\alpha, \beta, \gamma ::= \text{(lifetime variable)} \quad X, Y ::= \text{(type variable)}$$
$$x, y ::= \text{(variable)} \quad f, g ::= \text{(function name)} \quad L ::= \text{(label)}$$
$$\mathit{const} ::= n \mid () \quad \mathsf{bool} := \mathsf{unit} + \mathsf{unit} \quad \mathit{op} ::= \mathit{op}_{\mathsf{int}} \mid \mathit{op}_{\mathsf{bool}}$$
$$\mathit{op}_{\mathsf{int}} ::= + \mid - \mid \cdots \quad \mathit{op}_{\mathsf{bool}} ::= >= \mid == \mid != \mid \cdots$$

*Program, Function and Label.* A program (denoted by $\Pi$) is a set of function definitions. A function definition ($F$) consists of a function name, a function signature and a set of labeled statements ($L\colon S$). In COR, for simplicity, the input/output types of a function are restricted to *pointer types*. A function is parametrized over lifetime parameters under constraints; polymorphism on types is not supported for simplicity, just as $\lambda_{\mathsf{Rust}}$. For the lifetime parameter receiver, often $\langle \alpha_0, \cdots \mid \rangle$ is abbreviated to $\langle \alpha_0, \ldots \rangle$ and $\langle \mid \rangle$ is omitted.

A label ($L$) is an abstract program point to be jumped to by $\mathsf{goto}$.[10] Each label is assigned a *whole context* by the type system, as we see later. This style, with unstructured control flows, helps the formal description of CHCs in §3.2. A function should have the label $\mathsf{entry}$ (entry point), and every label in a function should be syntactically reachable from $\mathsf{entry}$ by $\mathsf{goto}$ jumps.[11]

*Statement and Instruction.* A statement ($S$) performs an instruction with a jump ($I$; $\mathsf{goto}\, L$), returns from a function ($\mathsf{return}\, x$), or branches ($\mathsf{match}\, {*x}\, \{\cdots\}$).

An instruction ($I$) performs an elementary operation: mutable (re)borrow ($\mathsf{let}\, y = \mathsf{mutbor}_\alpha\, x$), releasing a variable ($\mathsf{drop}\, x$), weakening ownership ($\mathsf{immut}\, x$),[12] swap ($\mathsf{swap}({*x}, {*y})$), creating/dereferencing a pointer ($\mathsf{let}\, {*y} = x$, $\mathsf{let}\, y = {*x}$), copy ($\mathsf{let}\, {*y} = \mathsf{copy}\, {*x}$),[13] type weakening ($x\ \mathsf{as}\ T$), function call ($\mathsf{let}\, y = f\langle\cdots\rangle(\cdots)$), lifetime-related ghost operations ($\mathsf{intro}\, \alpha$, $\mathsf{now}\, \alpha$, $\alpha \le \beta$; explained later), getting a constant / operation result / random integer ($\mathsf{let}\, {*y} = \mathit{const}$ / ${*x}\, \mathit{op} {*x'}$ / $\mathsf{rand}()$), creating a variant ($\mathsf{let}\, {*y} = \mathsf{inj}_i^{T_0+T_1}\, {*x}$), and creating/destructing a pair ($\mathsf{let}\, {*y} = ({*x_0}, {*x_1})$, $\mathsf{let}\, ({*y_0}, {*y_1}) = {*x}$). An instruction of form $\mathsf{let}\, {*y} = \cdots$ implicitly allocates new memory cells as $y$; also, some instructions deallocate memory cells implicitly. For simplicity, every variable is designed to be a *pointer* and every *release of a variable* should be explicitly annotated by '$\mathsf{drop}\, x$'. In addition, we provide swap instead of assignment; the usual assignment (of copyable data from ${*x}$ to ${*y}$) can be expressed by $\mathsf{let}\, {*x'} = \mathsf{copy}\, {*x};\ \mathsf{swap}({*y}, {*x'});\ \mathsf{drop}\, x'$.

*Type.* As a type ($T$), we support recursive types ($\mu X.T$), pointer types ($P\,T$), variant types ($T_0 + T_1$), pair types ($T_0 \times T_1$) and basic types ($\mathsf{int}, \mathsf{unit}$).

A pointer type $P\,T$ can be an *owning pointer* $\mathsf{own}\, T$ (`Box<T>` in Rust), *mutable reference* $\mathsf{mut}_\alpha\, T$ (`&'a mut T`) or *immutable reference* $\mathsf{immut}_\alpha\, T$ (`&'a T`). An

[10] It is related to a *continuation* introduced by $\mathsf{letcont}$ in $\lambda_{\mathsf{Rust}}$.

[11] Here 'syntactically' means that detailed information such that a branch condition on $\mathsf{match}$ or non-termination is ignored.

[12] This instruction turns a mutable reference to an immutable reference. Using this, an immutable borrow from $x$ to $y$ can be expressed by $\mathsf{let}\, y = \mathsf{mutbor}_\alpha\, x;\ \mathsf{immut}\, y$.

[13] Copying a pointer (an immutable reference) $x$ to $y$ can be expressed by $\mathsf{let}\, {*ox} = x;\ \mathsf{let}\, {*oy} = \mathsf{copy}\, {*ox};\ \mathsf{let}\, y = {*oy}$.

*owning pointer* has data in the heap memory, can freely update the data (unless it is borrowed), and has the obligation to clean up the data from the heap memory. In contrast, a *mutable/immutable reference* (or *unique/shared reference*) borrows an update/read permission from an owning pointer or another reference with the deadline of a *lifetime* $\alpha$ (introduced later). A mutable reference cannot be copied, while an immutable reference can be freely copied. A reference loses the permission at the time when it is released.[14]

A type $T$ that appears in a program (not just as a substructure of some type) should satisfy the following condition (if it holds we say the type is *complete*): every type variable $X$ in $T$ is bound by some $\mu$ and guarded by a pointer constructor (i.e. given a binding of form $\mu X.U$, every occurrence of $X$ in $U$ is a part of a pointer type, of form $P\,U'$).

*Lifetime.* A *lifetime* is an *abstract time point in the process of computation*,[15] which is statically managed by *lifetime variables* $\alpha$. A lifetime variable can be a *lifetime parameter* that a function takes or a *local lifetime variable* introduced within a function. We have three lifetime-related ghost instructions: $\mathsf{intro}\,\alpha$ introduces a new local lifetime variable, $\mathsf{now}\,\alpha$ sets a local lifetime variable to the current moment and eliminates it, and $\alpha \leq \beta$ asserts the ordering on local lifetime variables.

**Expressivity and Limitations.** COR can express most borrow patterns in the core of Rust. The set of moments when a borrow is active forms a continuous time range, even under *non-lexical lifetimes* [54].[16]

A major limitation of COR is that it does not support *unsafe code blocks* and also lacks *type traits and closures*. Still, our idea can be combined with unsafe code and closures, as discussed in §3.5. Another limitation of COR is that, unlike Rust and $\lambda_{\mathsf{Rust}}$, we *cannot directly modify/borrow a fragment of a variable* (e.g. an element of a pair). Still, we can eventually modify/borrow a fragment by borrowing the whole variable and *splitting pointers* (e.g. '$\mathsf{let}\,(*y_0, *y_1) = *x$'). This borrow-and-split strategy, nevertheless, yields a subtle obstacle when we extend the calculus for advanced data types (e.g. `get_default` in 'Problem Case #3' from [54]). For future work, we pursue a more expressive calculus modeling Rust and extend our verification method to it.

*Example 1 (COR Program).* The following program expresses the functions `take_max` and `inc_max` presented in § 1.2. We shorthand sequential executions

[14] In Rust, even after a reference loses the permission and the lifetime ends, its address data can linger in the memory, although dereferencing on the reference is no longer allowed. We simplify the behavior of lifetimes in COR.

[15] In the terminology of Rust, a lifetime often means a time range where a borrow is active. To simplify the discussions, however, we in this paper use the term lifetime to refer to a *time point when a borrow ends*.

[16] Strictly speaking, this property is broken by recently adopted implicit two-phase borrows [59,53]. However, by shallow syntactical reordering, a program with implicit two-phase borrows can be fit into usual borrow patterns.

by ‘$;^{L}$’ (e.g. $L_0{:}\, I_0;^{L_1} I_1;$ goto $L_2$ stands for $L_0{:}\, I_0;$ goto $L_1$ $L_1{:}\, I_1;$ goto $L_2$).[17]

```
fn take-max ⟨α⟩ (ma: mut_α int, mb: mut_α int) → mut_α int {
  entry: let *ord = *ma >= *mb;^L1 match *ord {inj_1 *ou → goto L2, inj_0 *ou → goto L5}
  L2: drop ou;^L3 drop mb;^L4 return ma    L5: drop ou;^L6 drop ma;^L7 return mb
}
fn inc-max(oa: own int, ob: own int) → own bool {
  entry: intro α;^L1 let ma = mutbor_α oa;^L2 let mb = mutbor_α ob;^L3
   let mc = take-max⟨α⟩(ma, mb);^L4 let *o1 = 1;^L5 let *oc' = *mc + *o1;^L6 drop o1;^L7
   swap(mc, oc');^L8 drop oc';^L9 drop mc;^L10 now α;^L11 let *or = *oa != *ob;^L12
   drop oa;^L13 drop ob;^L14 return or
}
```

In take-max, conditional branching is performed by match and its goto directions (at L1). In inc-max, increment on the mutable reference $mc$ is performed by calculating the new value (at L4, L5) and updating the data by swap (at L7).

The following is the corresponding Rust program, with ghost annotations (marked italic and dark green, e.g. *drop ma*) on lifetimes and releases of mutable references.

```
fn take_max<'a>(ma: &'a mut i32, mb: &'a mut i32) -> &'a mut i32 {
  if *ma >= *mb { drop mb; ma } else { drop ma; mb }
}
fn inc_max(mut a: i32, mut b: i32) -> bool {
  { intro 'a;
    let mc = take_max<'a>(&'a mut a, &'a mut b); *mc += 1;
  drop mc; now 'a; }
  a != b
}
```

#### 2.2 Type System

The type system of COR assigns to each label a *whole context* $(\mathbf{\Gamma}, \mathbf{A})$. We define below the whole context and the typing judgments.

*Context.* A *variable context* $\mathbf{\Gamma}$ is a finite set of items of form $x{:}^{\mathbf{a}}\, T$, where $T$ should be a complete *pointer* type and $\mathbf{a}$ (which we call *activeness*) is of form ‘active’ or ‘$\dagger\alpha$’ (*frozen* until lifetime $\alpha$). We abbreviate $x{:}^{\text{active}}\, T$ as $x{:}\, T$. A variable context should not contain two items on the same variable. A *lifetime context* $\mathbf{A} = (A, R)$ is a finite preordered set of lifetime variables, where $A$ is the underlying set and $R$ is the preorder. We write $|\mathbf{A}|$ and $\leq_{\mathbf{A}}$ to refer to $A$ and $R$. Finally, a *whole context* $(\mathbf{\Gamma}, \mathbf{A})$ is a pair of a variable context $\mathbf{\Gamma}$ and a lifetime context $\mathbf{A}$ such that every lifetime variable in $\mathbf{\Gamma}$ is contained in $\mathbf{A}$.

[17] The first character of each variable indicates the pointer kind ($o/m$ corresponds to own/mut$_\alpha$). We swap the branches of the match statement in take-max, to fit the order to C/Rust’s `if`.

*Notations.* The set operation $A + B$ (or more generally $\sum_\lambda A_\lambda$) denotes the disjoint union, i.e. the union defined only if the arguments are disjoint. The set operation $A - B$ denotes the set difference defined only if $A \supseteq B$. For a natural number $n$, $[n]$ denotes the set $\{0, \ldots, n-1\}$.

Generally, an auxiliary definition for a rule can be presented just below, possibly in a dotted box.

*Program and Function.* The rules for typing programs and functions are presented below. They assign to each label a whole context $(\mathbf{\Gamma}, \mathbf{A})$. '$S{:}_{\Pi,f}\,(\mathbf{\Gamma}, \mathbf{A}) \mid (\mathbf{\Gamma}_L, \mathbf{A}_L)_L \mid U$' is explained later.

$$\frac{\text{for any } F \text{ in } \Pi,\ F{:}_\Pi\,(\mathbf{\Gamma}_{\mathrm{name}(F),L}, \mathbf{A}_{\mathrm{name}(F),L})_{L\in\mathrm{Label}_F}}{\Pi{:}\,(\mathbf{\Gamma}_{f,L}, \mathbf{A}_{f,L})_{(f,L)\,\in\,\mathrm{FnLabel}_\Pi}}$$

name$(F)$: the function name of $F$ Label$_F$: the set of labels in $F$

FnLabel$_\Pi$: the set of pairs $(f, L)$ such that a function $f$ in $\Pi$ has a label $L$

$$\frac{\begin{array}{c} F = \mathsf{fn}\, f\langle \alpha_0, \ldots, \alpha_{m-1} \,|\, \alpha_{a_0} \le \alpha_{b_0}, \ldots, \alpha_{a_{l-1}} \le \alpha_{b_{l-1}} \rangle (x_0{:}\,T_0, \ldots, x_{n-1}{:}\,T_{n-1}) \to U\,\{\cdots\} \\ \mathbf{\Gamma}_{\mathsf{entry}} = \{x_i{:}\,T_i \,|\, i \in [n]\} \quad A = \{\alpha_j \,|\, j \in [m]\} \quad \mathbf{A}_{\mathsf{entry}} = \big(A, \big(\mathrm{Id}_A \cup \{(\alpha_{a_k}, \alpha_{b_k}) \,|\, k \in [l]\}\big)^+\big) \\ \text{for any } L'{:}\,S \in \mathrm{LabelStmt}_F,\ S{:}_{\Pi,f}\,(\mathbf{\Gamma}_{L'}, \mathbf{A}_{L'}) \mid (\mathbf{\Gamma}_L, \mathbf{A}_L)_{L\in\mathrm{Label}_F} \mid U \end{array}}{F{:}_\Pi\ \ (\mathbf{\Gamma}_L, \mathbf{A}_L)_{L\in\mathrm{Label}_F}}$$

LabelStmt$_F$: the set of labeled statements in $F$

Id$_A$: the identity relation on $A$ $R^+$: the transitive closure of $R$

On the rule for the function, the initial whole context at $\mathsf{entry}$ is specified (the second and third preconditions) and also the contexts for other labels are checked (the fourth precondition). The context for each label (in each function) can actually be determined in the order by the distance in the number of $\mathsf{goto}$ jumps from $\mathsf{entry}$, but that order is not very obvious because of *unstructured control flows*.

*Statement.* '$S{:}_{\Pi,f}\,(\mathbf{\Gamma}, \mathbf{A}) \mid (\mathbf{\Gamma}_L, \mathbf{A}_L)_L \mid U$' means that running the statement $S$ (under $\Pi, f$) with the whole context $(\mathbf{\Gamma}, \mathbf{A})$ results in a jump to a label with the whole contexts specified by $(\mathbf{\Gamma}_L, \mathbf{A}_L)_L$ or a return of data of type $U$. Its rules are presented below. '$I{:}_{\Pi,f}\,(\mathbf{\Gamma}, \mathbf{A}) \to (\mathbf{\Gamma}', \mathbf{A}')$' is explained later.

$$\frac{I{:}_{\Pi,f}\,(\mathbf{\Gamma}, \mathbf{A}) \to (\mathbf{\Gamma}_{L_0}, \mathbf{A}_{L_0})}{I;\, \mathsf{goto}\, L_0{:}_{\Pi,f}\,(\mathbf{\Gamma}, \mathbf{A}) \mid (\mathbf{\Gamma}_L, \mathbf{A}_L)_L \mid U} \qquad \frac{\mathbf{\Gamma} = \{x{:}\,U\} \quad |\mathbf{A}| = A_{\mathrm{ex}\,\Pi,f}}{\mathsf{return}\, x{:}_{\Pi,f}\,(\mathbf{\Gamma}, \mathbf{A}) \mid (\mathbf{\Gamma}_L, \mathbf{A}_L)_L \mid U}$$

$A_{\mathrm{ex}\,\Pi,f}$: the set of lifetime parameters of $f$ in $\Pi$

$$\frac{\begin{array}{c} x{:}\,P\,(T_0{+}T_1) \in \mathbf{\Gamma} \\ \text{for } i = 0, 1,\ (\mathbf{\Gamma}_{L_i}, \mathbf{A}_{L_i}) = (\mathbf{\Gamma} - \{x{:}\,P\,(T_0{+}T_1)\} + \{y_i{:}\,P\,T_i\},\ \mathbf{A}) \end{array}}{\mathsf{match} * x\,\{\mathsf{inj}_0 * y_0 \to \mathsf{goto}\, L_0,\ \mathsf{inj}_1 * y_1 \to \mathsf{goto}\, L_1\}{:}_{\Pi,f}\,(\mathbf{\Gamma}, \mathbf{A}) \mid (\mathbf{\Gamma}_L, \mathbf{A}_L)_L \mid U}$$

The rule for the $\mathsf{return}$ statement ensures that there remain no extra variables and local lifetime variables.

*Instruction.* '$I{:}_{\Pi,f}\,(\mathbf{\Gamma}, \mathbf{A}) \to (\mathbf{\Gamma}', \mathbf{A}')$' means that running the instruction $I$ (under $\Pi, f$) updates the whole context $(\mathbf{\Gamma}, \mathbf{A})$ into $(\mathbf{\Gamma}', \mathbf{A}')$. The rules are designed so that, for any $I$, $\Pi$, $f$, $(\mathbf{\Gamma}, \mathbf{A})$, there exists at most one $(\mathbf{\Gamma}', \mathbf{A}')$ such that

$I \colon_{\Pi,f} (\mathbf{\Gamma}, \mathbf{A}) \to (\mathbf{\Gamma}', \mathbf{A}')$ holds. Below we present some of the rules; the complete rules are presented in the full paper. The following is the typing rule for mutable (re)borrow.

$$\frac{\alpha \notin A_{\mathrm{ex}\,\Pi,f} \quad P = \mathsf{own}, \mathsf{mut}_\alpha \quad \text{for any } \beta \in \mathrm{Lifetime}_{P\,T},\ \alpha \leq_{\mathbf{A}} \beta}{\mathsf{let}\, y = \mathsf{mutbor}_\alpha\, x \colon_{\Pi,f} (\mathbf{\Gamma} + \{x \colon P\,T\},\ \mathbf{A}) \to (\mathbf{\Gamma} + \{y \colon \mathsf{mut}_\alpha\, T,\ x \colon^{\dagger\alpha} P\,T\},\ \mathbf{A})}$$

$$\mathrm{Lifetime}_T\text{: the set of lifetime variables occurring in } T$$

After you mutably (re)borrow an owning pointer / mutable reference $x$ until $\alpha$, $x$ is *frozen* until $\alpha$. Here, $\alpha$ should be a local lifetime variable[18] (the first precondition) that does not live longer than the data of $x$ (the third precondition). Below are the typing rules for local lifetime variable introduction and elimination.

$$\mathsf{intro}\,\alpha \colon_{\Pi,f} \big(\mathbf{\Gamma}, (A, R)\big) \to \big(\mathbf{\Gamma}, (\{\alpha\} + A,\ \{\alpha\} \times (\{\alpha\} + A_{\mathrm{ex}\,\Pi,f}) + R)\big)$$

$$\frac{\alpha \notin A_{\mathrm{ex}\,\Pi,f}}{\mathsf{now}\,\alpha \colon_{\Pi,f} \big(\mathbf{\Gamma}, (\{\alpha\} + A,\ R)\big) \to \big(\{\mathrm{thaw}_\alpha(x \colon^{\mathbf{a}} T) \mid x \colon^{\mathbf{a}} T \in \mathbf{\Gamma}\},\ (A, \{(\beta, \gamma) \in R \mid \beta \neq \alpha\})\big)}$$

$$\mathrm{thaw}_\alpha(x \colon^{\mathbf{a}} T) := \begin{cases} x \colon T & (\mathbf{a} = \dagger\alpha) \\ x \colon^{\mathbf{a}} T & (\text{otherwise}) \end{cases}$$

On $\mathsf{intro}\,\alpha$, it just ensures the new local lifetime variable to be earlier than any lifetime parameters (which are given by exterior functions). On $\mathsf{now}\,\alpha$, the variables frozen with $\alpha$ get active again. Below is the typing rule for dereference of a pointer to a pointer, which may be a bit interesting.

$$\mathsf{let}\, y = *x \colon_{\Pi,f} (\mathbf{\Gamma} + \{x \colon P\,P'\,T\},\ \mathbf{A}) \to (\mathbf{\Gamma} + \{y \colon (P \circ P')\,T\},\ \mathbf{A})$$

$$P \circ \mathsf{own} = \mathsf{own} \circ P := P \qquad R_\alpha \circ R'_\beta := R''_\alpha \text{ where } R'' = \begin{cases} \mathsf{mut} & (R = R' = \mathsf{mut}) \\ \mathsf{immut} & (\text{otherwise}) \end{cases}$$

The third precondition of the typing rule for $\mathsf{mutbor}$ justifies taking just $\alpha$ in the rule '$R_\alpha \circ R'_\beta := R''_\alpha$'.

Let us interpret $\Pi \colon (\mathbf{\Gamma}_{f,L}, \mathbf{A}_{f,L})_{(f,L) \in \mathrm{FnLabel}_\Pi}$ as "the program $\Pi$ has the type $(\mathbf{\Gamma}_{f,L}, \mathbf{A}_{f,L})_{(f,L) \in \mathrm{FnLabel}_\Pi}$". The type system ensures that any program has at most one type (which may be a bit unclear because of unstructured control flows). Hereinafter, we implicitly assume that a program has a type.

#### 2.3 Concrete Operational Semantics

We introduce for COR *concrete operational semantics*, which handles a concrete model of the heap memory.

The basic item, *concrete configuration* $\mathbf{C}$, is defined as follows.

$$\mathbf{S} ::= \mathrm{end} \ \big|\ [f, L]\, x, \mathbf{F};\, \mathbf{S} \quad (\text{concrete configuration})\ \mathbf{C} ::= [f, L]\, \mathbf{F};\, \mathbf{S} \mid \mathbf{H}$$

Here, $\mathbf{H}$ is a *heap*, which maps addresses (represented by integers) to integers (data). $\mathbf{F}$ is a *concrete stack frame*, which maps variables to addresses. The stack

[18] In COR, a reference that lives after the return from the function should be created by splitting a reference (e.g. '$\mathsf{let}\,(*y_0, *y_1) = *x$') given in the inputs; see also Expressivity and Limitations.

part of $\mathbf{C}$ is of form '$[f, L]\,\mathbf{F};\ [f', L']\,x, \mathbf{F}';\ \cdots;$ end' (we may omit the terminator '; end'). $[f, L]$ on each stack frame indicates the program point. '$x$,' on each non-top stack frame is the receiver of the value returned by the function call.

Concrete operational semantics is characterized by the one-step transition relation $\mathbf{C} \to_\Pi \mathbf{C}'$ and the termination relation $\mathrm{final}_\Pi(\mathbf{C})$, which can be defined straightforwardly. Below we show the rules for mutable (re)borrow, swap, function call and return from a function; the complete rules and an example execution are presented in the full paper. $S_{\Pi,f,L}$ is the statement for the label $L$ of the function $f$ in $\Pi$. $\mathrm{Ty}_{\Pi,f,L}(x)$ is the type of variable $x$ at the label.

$$\frac{S_{\Pi,f,L} = \mathsf{let}\, y = \mathsf{mutbor}_\alpha\, x;\ \mathsf{goto}\, L' \quad \mathbf{F}(x) = a}{[f, L]\,\mathbf{F};\, \mathbf{S} \mid \mathbf{H} \ \to_\Pi\ [f, L']\,\mathbf{F}{+}\{(y, a)\};\, \mathbf{S} \mid \mathbf{H}}$$

$$\frac{S_{\Pi,f,L} = \mathsf{swap}(*x, *y);\ \mathsf{goto}\, L' \quad \mathrm{Ty}_{\Pi,f,L}(x) = P\,T \quad \mathbf{F}(x) = a \quad \mathbf{F}(y) = b}{\begin{array}{l}[f, L]\,\mathbf{F};\, \mathbf{S} \mid \mathbf{H}{+}\{(a{+}k, m_k) \mid k \in [\#T]\}{+}\{(b{+}k, n_k) \mid k \in [\#T]\} \\ \quad \to_\Pi\ [f, L']\,\mathbf{F};\, \mathbf{S} \mid \mathbf{H}{+}\{(a{+}k, n_k) \mid k \in [\#T]\}{+}\{(b{+}k, m_k) \mid k \in [\#T]\}\end{array}}$$

$$\frac{\begin{array}{c}S_{\Pi,f,L} = \mathsf{let}\, y = g\langle\cdots\rangle(x_0, \ldots, x_{n-1});\ \mathsf{goto}\, L' \\ \Sigma_{\Pi,g} = \langle\cdots\rangle(x'_0{:}\,T_0, \ldots, x'_{n-1}{:}\,T_{n-1}) \to U\end{array}}{[f, L]\,\mathbf{F}{+}\{(x_i, a_i) \mid i \in [n]\};\, \mathbf{S} \mid \mathbf{H} \to_\Pi\ [g, \mathsf{entry}]\,\{(x'_i, a_i) \mid i \in [n]\};\ [f, L]\,y, \mathbf{F};\, \mathbf{S} \mid \mathbf{H}}$$

$$\frac{S_{\Pi,f,L} = \mathsf{return}\, x}{[f, L]\,\{(x, a)\}; [g, L']\,x', \mathbf{F}'; \mathbf{S} \mid \mathbf{H} \to_\Pi [g, L']\,\mathbf{F}'{+}\{(x', a)\}; \mathbf{S} \mid \mathbf{H}}$$

$$\frac{S_{\Pi,f,L} = \mathsf{return}\, x}{\mathrm{final}_\Pi\big([f, L]\,\{(x, a)\} \mid \mathbf{H}\big)}$$

Here we introduce '$\#T$', which represents how many memory cells the type $T$ takes (at the outermost level). $\#T$ is defined for every *complete* type $T$, because every occurrence of type variables in a complete type is guarded by a pointer constructor.

$$\#(T_0{+}T_1) := 1 + \max\{\#T_0, \#T_1\} \quad \#(T_0{\times}T_1) := \#T_0 + \#T_1$$
$$\#\,\mu X.T := \#\,T[\mu X.T/X] \quad \#\,\mathsf{int} = \#\,P\,T := 1 \quad \#\,\mathsf{unit} = 0$$

## 3 CHC Representation of COR Programs

To formalize the idea discussed in § 1, we give a translation from COR programs to CHC systems, which precisely characterize the input-output relations of the COR programs. We first define the logic for CHCs (§ 3.1). We then formally describe our translation (§ 3.2) and prove its correctness (§ 3.3). Also, we examine effectiveness of our approach with advanced examples (§ 3.4) and discuss how our idea can be extended and enhanced (§ 3.5).

### 3.1 Multi-sorted Logic for Describing CHCs

To begin with, we introduce a first-order multi-sorted logic for describing the CHC representation of COR programs.

**Syntax.** The syntax is defined as follows.

$$
\begin{gathered}
\text{(CHC) } \Phi \ ::= \ \forall x_0\colon \sigma_0, \dots, x_{m-1}\colon \sigma_{m-1}.\ \check{\varphi} \ \Longleftarrow\ \psi_0 \wedge \cdots \wedge \psi_{n-1} \\
\top := \text{the nullary conjunction of formulas} \\
\text{(formula) } \varphi, \psi \ ::= \ f(t_0, \dots, t_{n-1}) \quad \text{(elementary formula) } \check{\varphi} \ ::= \ f(p_0, \dots, p_{n-1}) \\
\text{(term) } t \ ::= \ x \mid \langle t \rangle \mid \langle t_*, t_\circ \rangle \mid \mathsf{inj}_i\, t \mid (t_0, t_1) \mid *t \mid \circ t \mid t.i \mid \mathit{const} \mid t\ \mathit{op}\ t' \\
\text{(value) } v, w \ ::= \ \langle v \rangle \mid \langle v_*, v_\circ \rangle \mid \mathsf{inj}_i\, v \mid (v_0, v_1) \mid \mathit{const} \\
\text{(pattern) } p, q \ ::= \ x \mid \langle p \rangle \mid \langle p_*, p_\circ \rangle \mid \mathsf{inj}_i\, p \mid (p_0, p_1) \mid \mathit{const} \\
\text{(sort) } \sigma, \tau \ ::= \ X \mid \mu X.\sigma \mid C\,\sigma \mid \sigma_0 + \sigma_1 \mid \sigma_0 \times \sigma_1 \mid \mathsf{int} \mid \mathsf{unit} \\
\text{(container kind) } C \ ::= \ \mathsf{box} \mid \mathsf{mut} \quad \mathit{const} \ ::= \ \text{same as COR} \quad \mathit{op} \ ::= \ \text{same as COR} \\
\mathsf{bool} := \mathsf{unit} + \mathsf{unit} \quad \mathsf{true} := \mathsf{inj}_1\,() \quad \mathsf{false} := \mathsf{inj}_0\,() \\
X \ ::= \ \text{(sort variable)} \quad x, y \ ::= \ \text{(variable)} \quad f \ ::= \ \text{(predicate variable)}
\end{gathered}
$$

We introduce $\mathsf{box}\,\sigma$ and $\mathsf{mut}\,\sigma$, which correspond to $\mathsf{own}\,T/\mathsf{immut}_\alpha\,T$ and $\mathsf{mut}_\alpha\,T$ respectively. $\langle t \rangle/\langle t_*, t_\circ \rangle$ is the constructor for $\mathsf{box}\,\sigma/\mathsf{mut}\,\sigma$. $*t$ takes the body/first value of $\langle - \rangle/\langle -, - \rangle$ and $\circ t$ takes the second value of $\langle -, - \rangle$. We restrict the form of CHCs here to simplify the proofs later. Although the logic does not have a primitive for equality, we can define the equality in a CHC system (e.g. by adding $\forall x\colon \sigma.\, Eq(x, x) \ \Longleftarrow\ \top$).

A *CHC system* $(\mathbf{\Phi}, \mathbf{\Xi})$ is a pair of a finite set of CHCs $\mathbf{\Phi} = \{\Phi_0, \dots, \Phi_{n-1}\}$ and $\mathbf{\Xi}$, where $\mathbf{\Xi}$ is a finite map from predicate variables to tuples of sorts (denoted by $\Xi$), specifying the sorts of the input values. Unlike the informal description in § 1, we add $\mathbf{\Xi}$ to a CHC system.

**Sort System.** '$t{:}_{\mathbf{\Delta}}\, \sigma$' (the term $t$ has the sort $\sigma$ under $\mathbf{\Delta}$) is defined as follows. Here, $\mathbf{\Delta}$ is a finite map from variables to sorts. $\sigma \sim \tau$ is the congruence on sorts induced by $\mu X.\sigma \sim \sigma[\mu X.\sigma/X]$.

$$
\frac{\mathbf{\Delta}(x) = \sigma}{x{:}_{\mathbf{\Delta}}\, \sigma} \quad
\frac{t{:}_{\mathbf{\Delta}}\, \sigma}{\langle t \rangle{:}_{\mathbf{\Delta}}\, \mathsf{box}\,\sigma} \quad
\frac{t_*, t_\circ{:}_{\mathbf{\Delta}}\, \sigma}{\langle t_*, t_\circ \rangle{:}_{\mathbf{\Delta}}\, \mathsf{mut}\,\sigma} \quad
\frac{t{:}_{\mathbf{\Delta}}\, \sigma_i}{\mathsf{inj}_i\, t{:}_{\mathbf{\Delta}}\, \sigma_0 + \sigma_1} \quad
\frac{t_0{:}_{\mathbf{\Delta}}\, \sigma_0 \quad t_1{:}_{\mathbf{\Delta}}\, \sigma_1}{(t_0, t_1){:}_{\mathbf{\Delta}}\, \sigma_0 \times \sigma_1}
$$

$$
\frac{t{:}_{\mathbf{\Delta}}\, C\,\sigma}{*t{:}_{\mathbf{\Delta}}\, \sigma} \quad
\frac{t{:}_{\mathbf{\Delta}}\, \mathsf{mut}\,\sigma}{\circ t{:}_{\mathbf{\Delta}}\, \sigma} \quad
\frac{t{:}_{\mathbf{\Delta}}\, \sigma_0 + \sigma_1}{t.i{:}_{\mathbf{\Delta}}\, \sigma_i} \quad
\mathit{const}{:}_{\mathbf{\Delta}}\, \sigma_{\mathit{const}} \quad
\frac{t, t'{:}_{\mathbf{\Delta}}\, \mathsf{int}}{t\ \mathit{op}\ t'{:}_{\mathbf{\Delta}}\, \sigma_{\mathit{op}}} \quad
\frac{t{:}_{\mathbf{\Delta}}\, \sigma \quad \sigma \sim \tau}{t{:}_{\mathbf{\Delta}}\, \tau}
$$

$\sigma_{\mathit{const}}$: the sort of *const* $\quad \sigma_{\mathit{op}}$: the output sort of *op*

'wellSorted$_{\mathbf{\Delta},\mathbf{\Xi}}(\varphi)$' and 'wellSorted$_{\mathbf{\Xi}}(\mathbf{\Phi})$', the judgments on well-sortedness of formulas and CHCs, are defined as follows.

$$
\frac{\mathbf{\Xi}(f) = (\sigma_0, \dots, \sigma_{n-1}) \quad \text{for any } i \in [n],\ t_i{:}_{\mathbf{\Delta}}\, \sigma_i}{\text{wellSorted}_{\mathbf{\Delta},\mathbf{\Xi}}(f(t_0, \dots, t_{n-1}))}
$$

$$
\frac{\mathbf{\Delta} = \{(x_i, \sigma_i) \mid i \in [m]\} \quad \text{wellSorted}_{\mathbf{\Delta},\mathbf{\Xi}}(\check{\varphi}) \quad \text{for any } j \in [n],\ \text{wellSorted}_{\mathbf{\Delta},\mathbf{\Xi}}(\psi_j)}{\text{wellSorted}_{\mathbf{\Xi}}\big(\forall x_0\colon \sigma_0, \dots, x_{m-1}\colon \sigma_{m-1}.\ \check{\varphi} \ \Longleftarrow\ \psi_0 \wedge \cdots \wedge \psi_{n-1}\big)}
$$

The CHC system $(\mathbf{\Phi}, \mathbf{\Xi})$ is said to be well-sorted if wellSorted$_{\mathbf{\Xi}}(\Phi)$ holds for any $\Phi \in \mathbf{\Phi}$.

**Semantics.** '$[\![t]\!]_{\mathbf{I}}$', the interpretation of the term $t$ as a value under $\mathbf{I}$, is defined as follows. Here, $\mathbf{I}$ is a finite map from variables to values. Although the definition is partial, the interpretation is defined for all well-sorted terms.

$$[\![x]\!]_{\mathbf{I}} := \mathbf{I}(x) \quad [\![\langle t\rangle]\!]_{\mathbf{I}} := \langle [\![t]\!]_{\mathbf{I}}\rangle \quad [\![\langle t_*, t_\circ\rangle]\!]_{\mathbf{I}} := \langle [\![t_*]\!]_{\mathbf{I}}, [\![t_\circ]\!]_{\mathbf{I}}\rangle \quad [\![\mathsf{inj}_i\, t]\!]_{\mathbf{I}} := \mathsf{inj}_i [\![t]\!]_{\mathbf{I}}$$

$$[\![(t_0, t_1)]\!]_{\mathbf{I}} := ([\![t_0]\!]_{\mathbf{I}}, [\![t_1]\!]_{\mathbf{I}}) \quad [\![*t]\!]_{\mathbf{I}} := \begin{cases} v & ([\![t]\!]_{\mathbf{I}} = \langle v\rangle) \\ v_* & ([\![t]\!]_{\mathbf{I}} = \langle v_*, v_\circ\rangle) \end{cases} \quad [\![\circ t]\!]_{\mathbf{I}} := v_\circ \text{ if } [\![t]\!]_{\mathbf{I}} = \langle v_*, v_\circ\rangle$$

$$[\![t.i]\!]_{\mathbf{I}} := v_i \text{ if } [\![t]\!]_{\mathbf{I}} = (v_0, v_1) \quad [\![\mathit{const}]\!]_{\mathbf{I}} := \mathit{const} \quad [\![t\ \mathit{op}\ t']\!]_{\mathbf{I}} := [\![t]\!]_{\mathbf{I}} [\![\mathit{op}]\!] [\![t']\!]_{\mathbf{I}}$$

$[\![\mathit{op}]\!]$: the binary operation on values corresponding to $\mathit{op}$

A *predicate structure* $\mathbf{M}$ is a finite map from predicate variables to (concrete) predicates on values. $\mathbf{M}, \mathbf{I} \models f(t_0, \dots, t_{n-1})$ means that $\mathbf{M}(f)([\![t_0]\!]_{\mathbf{I}}, \dots, [\![t_{m-1}]\!]_{\mathbf{I}})$ holds. $\mathbf{M} \models \Phi$ is defined as follows.

$$\frac{\text{for any } \mathbf{I} \text{ s.t. } \forall i \in [m].\, \mathbf{I}(x_i) {:}_{\varnothing}\, \sigma_i,\ \mathbf{M}, \mathbf{I} \models \psi_0, \dots, \psi_{n-1} \text{ implies } \mathbf{M}, \mathbf{I} \models \check{\varphi}}{\mathbf{M} \models \forall x_0{:}\,\sigma_0, \dots, x_{m-1}{:}\,\sigma_{m-1}.\ \ \check{\varphi} \ \Longleftarrow\ \psi_0 \wedge \cdots \wedge \psi_{n-1}}$$

Finally, $\mathbf{M} \models (\mathbf{\Phi}, \mathbf{\Xi})$ is defined as follows.

$$\frac{\begin{array}{c}\text{for any } (f, (\sigma_0, \dots, \sigma_{n-1})) \in \mathbf{\Xi},\ \mathbf{M}(f) \text{ is a predicate on values of sort } \sigma_0, \dots, \sigma_{n-1} \\ \operatorname{dom} \mathbf{M} = \operatorname{dom} \mathbf{\Xi} \quad \text{for any } \Phi \in \mathbf{\Phi},\ \mathbf{M} \models \Phi\end{array}}{\mathbf{M} \models (\mathbf{\Phi}, \mathbf{\Xi})}$$

When $\mathbf{M} \models (\mathbf{\Phi}, \mathbf{\Xi})$ holds, we say that $\mathbf{M}$ is a *model* of $(\mathbf{\Phi}, \mathbf{\Xi})$. Every well-sorted CHC system $(\mathbf{\Phi}, \mathbf{\Xi})$ has the *least model* on the point-wise ordering (which can be proved based on the discussions in [16]), which we write as $\mathbf{M}^{\text{least}}_{(\mathbf{\Phi}, \mathbf{\Xi})}$.

#### 3.2 Translation from COR Programs to CHCs

Now we formalize our translation of Rust programs into CHCs. We define $(\!|\Pi|\!)$, which is a CHC system that represents the input-output relations of the functions in the COR program $\Pi$.

Roughly speaking, the least model $\mathbf{M}^{\text{least}}_{(\!|\Pi|\!)}$ for this CHC system should satisfy: for any values $v_0, \dots, v_{n-1}, w$, $\mathbf{M}^{\text{least}}_{(\!|\Pi|\!)} \models f_{\mathsf{entry}}(v_0, \dots, v_{n-1}, w)$ holds exactly if, in COR, a function call $f(v_0, \dots, v_{n-1})$ can return $w$. Actually, in concrete operational semantics, such values should be read out from the heap memory. The formal description and proof of this expected property is presented in §3.3.

**Auxiliary Definitions.** The sort corresponding to the type $T$, $(\!|T|\!)$, is defined as follows. $\check{P}$ is a meta-variable for a non-mutable-reference pointer kind, i.e. $\mathsf{own}$ or $\mathsf{immut}_\alpha$. Note that the information on lifetimes is all stripped off.

$$(\!|X|\!) := X \quad (\!|\mu X.T|\!) = \mu X.(\!|T|\!) \quad (\!|\check{P}\, T|\!) := \mathsf{box}\, (\!|T|\!) \quad (\!|\mathsf{mut}_\alpha\, T|\!) := \mathsf{mut}\, (\!|T|\!)$$

$$(\!|\mathsf{int}|\!) := \mathsf{int} \quad (\!|\mathsf{unit}|\!) := \mathsf{unit} \quad (\!|T_0 {+} T_1|\!) := (\!|T_0|\!) + (\!|T_1|\!) \quad (\!|T_0 {\times} T_1|\!) := (\!|T_0|\!) \times (\!|T_1|\!)$$

We introduce a special variable $\mathsf{res}$ to represent the result of a function.[19] For a label $L$ in a function $f$ in a program $\Pi$, we define $\check{\varphi}_{\Pi,f,L}$, $\Xi_{\Pi,f,L}$ and $\mathbf{\Delta}_{\Pi,f,L}$

[19] For simplicity, we assume that the parameters of each function are sorted respecting *some fixed order* on variables (with $\mathsf{res}$ coming at the last), and we enumerate various items in this fixed order.

as follows, if the items in the variable context for the label are enumerated as $x_0{:}^{\mathbf{a}_0} T_0, \ldots, x_{n-1}{:}^{\mathbf{a}_{n-1}} T_{n-1}$ and the return type of the function is $U$.

$$\check{\varphi}_{\Pi,f,L} := f_L(x_0, \ldots, x_{n-1}, \mathsf{res}) \quad \Xi_{\Pi,f,L} := ((\!|T_0|\!), \ldots, (\!|T_{n-1}|\!), (\!|U|\!))$$
$$\mathbf{\Delta}_{\Pi,f,L} := \{(x_i, (\!|T_i|\!)) \mid i \in [n]\} + \{(\mathsf{res}, (\!|U|\!))\}$$

$\forall(\mathbf{\Delta})$ stands for $\forall\, x_0{:}\, \sigma_0, \ldots, x_{n-1}{:}\, \sigma_{n-1}$, where the items in $\mathbf{\Delta}$ are enumerated as $(x_0, \sigma_0), \ldots, (x_{n-1}, \sigma_{n-1})$.

**CHC Representation.** Now we introduce '$(\!|L{:}\, S|\!)_{\Pi,f}$', the set (in most cases, singleton) of CHCs modeling the computation performed by the labeled statement $L{:}\, S$ in $f$ from $\Pi$. Unlike informal descriptions in § 1, we turn to *pattern matching* instead of equations, to simplify the proofs. Below we show some of the rules; the complete rules are presented in the full paper. The variables marked green (e.g. $x_\circ$) should be fresh. The following is the rule for mutable (re)borrow.

$$(\!|L{:}\, \mathsf{let}\, y = \mathsf{mutbor}_\alpha\, x;\ \mathsf{goto}\, L'|\!)_{\Pi,f}$$
$$:= \begin{cases} \left\{ \begin{array}{l} \forall(\mathbf{\Delta}_{\Pi,f,L} + \{(x_\circ, (\!|T|\!))\}). \\ \quad \check{\varphi}_{\Pi,f,L} \impliedby \check{\varphi}_{\Pi,f,L'}[\langle *x, x_\circ\rangle/y, \langle x_\circ\rangle/x] \end{array} \right\} & (\mathrm{Ty}_{\Pi,f,L}(x) = \mathsf{own}\, T) \\ \left\{ \begin{array}{l} \forall(\mathbf{\Delta}_{\Pi,f,L} + \{(x_\circ, (\!|T|\!))\}). \\ \quad \check{\varphi}_{\Pi,f,L} \impliedby \check{\varphi}_{\Pi,f,L'}[\langle *x, x_\circ\rangle/y, \langle x_\circ, \circ x\rangle/x] \end{array} \right\} & (\mathrm{Ty}_{\Pi,f,L}(x) = \mathsf{mut}_\alpha\, T) \end{cases}$$

The value at the end of borrow is represented as a newly introduced variable $x_\circ$. Below is the rule for release of a variable.

$$(\!|L{:}\, \mathsf{drop}\, x;\ \mathsf{goto}\, L'|\!)_{\Pi,f}$$
$$:= \begin{cases} \left\{ \forall(\mathbf{\Delta}_{\Pi,f,L}).\ \check{\varphi}_{\Pi,f,L} \impliedby \check{\varphi}_{\Pi,f,L'} \right\} & (\mathrm{Ty}_{\Pi,f,L}(x) = \check{P}\, T) \\ \left\{ \begin{array}{l} \forall(\mathbf{\Delta}_{\Pi,f,L} - \{(x, \mathsf{mut}\, (\!|T|\!))\} + \{(x_*, (\!|T|\!))\}). \\ \quad \check{\varphi}_{\Pi,f,L}[\langle x_*, x_*\rangle/x] \impliedby \check{\varphi}_{\Pi,f,L'} \end{array} \right\} & (\mathrm{Ty}_{\Pi,f,L}(x) = \mathsf{mut}_\alpha\, T) \end{cases}$$

When a variable $x$ of type $\mathsf{mut}_\alpha\, T$ is dropped/released, we check the prophesied value at the end of borrow. Below is the rule for a function call.

$$(\!|L{:}\, \mathsf{let}\, y = g\langle\cdots\rangle(x_0, \ldots, x_{n-1});\ \mathsf{goto}\, L'|\!)_{\Pi,f}$$
$$:= \ \{\forall(\mathbf{\Delta}_{\Pi,f,L} + \{(y, (\!|\mathrm{Ty}_{\Pi,f,L'}(y)|\!))\}).\ \check{\varphi}_{\Pi,f,L} \impliedby g_{\mathsf{entry}}(x_0, \ldots, x_{n-1}, y) \wedge \check{\varphi}_{\Pi,f,L'}\}$$

The body (the right-hand side of $\impliedby$) of the CHC contains two formulas, which yields a kind of call stack at the level of CHCs. Below is the rule for a return from a function.

$$(\!|L{:}\, \mathsf{return}\, x|\!)_{\Pi,f} \ := \ \left\{ \forall(\mathbf{\Delta}_{\Pi,f,L}).\ \check{\varphi}_{\Pi,f,L}[x/\mathsf{res}] \impliedby \top \right\}$$

The variable $\mathsf{res}$ is forced to be equal to the returned variable $x$.

Finally, $(\!|\Pi|\!)$, the CHC system that represents the COR program $\Pi$ (or the *CHC representation* of $\Pi$), is defined as follows.

$$(\!|\Pi|\!) := \left(\textstyle\sum_{F \text{ in } \Pi,\ L:S\, \in\, \mathrm{LabelStmt}_F} (\!|L{:}\, S|\!)_{\Pi,\mathrm{name}_F},\ (\Xi_{\Pi,f,L})_{f_L \text{ s.t. } (f,L)\, \in\, \mathrm{FnLabel}_\Pi}\right)$$

*Example 2 (CHC Representation).* We present below the CHC representation of take-max described in § 2.1. We omit CHCs on inc-max here. We have also excluded the variable binders '$\forall\,\cdots$'.[20]

$$\mathsf{take\text{-}max}_{\mathsf{entry}}(ma, mb, \mathsf{res}) \impliedby \mathsf{take\text{-}max}_{\mathsf{L1}}(ma, mb, \langle *ma{>=}*mb\rangle, \mathsf{res})$$

[20] The sorts of the variables are as follows: $ma, mb, \mathsf{res}{:}\ \mathsf{mut\,int}$; $ma_*, mb_*{:}\ \mathsf{int}$; $ou{:}\ \mathsf{box\,unit}$.

$$\begin{aligned}
\mathsf{take\text{-}max}_{\mathsf{L1}}(ma, mb, \langle \mathsf{inj}_1 {*ou}\rangle, \mathsf{res}) &\Longleftarrow \mathsf{take\text{-}max}_{\mathsf{L2}}(ma, mb, ou, \mathsf{res}) \\
\mathsf{take\text{-}max}_{\mathsf{L1}}(ma, mb, \langle \mathsf{inj}_0 {*ou}\rangle, \mathsf{res}) &\Longleftarrow \mathsf{take\text{-}max}_{\mathsf{L5}}(ma, mb, ou, \mathsf{res}) \\
\mathsf{take\text{-}max}_{\mathsf{L2}}(ma, mb, ou, \mathsf{res}) &\Longleftarrow \mathsf{take\text{-}max}_{\mathsf{L3}}(ma, mb, \mathsf{res}) \\
\mathsf{take\text{-}max}_{\mathsf{L3}}(ma, \langle mb_*, mb_*\rangle, \mathsf{res}) &\Longleftarrow \mathsf{take\text{-}max}_{\mathsf{L4}}(ma, \mathsf{res}) \\
\mathsf{take\text{-}max}_{\mathsf{L4}}(ma, ma) &\Longleftarrow \top \\
\mathsf{take\text{-}max}_{\mathsf{L5}}(ma, mb, ou, \mathsf{res}) &\Longleftarrow \mathsf{take\text{-}max}_{\mathsf{L6}}(ma, mb, \mathsf{res}) \\
\mathsf{take\text{-}max}_{\mathsf{L6}}(\langle ma_*, ma_*\rangle, mb, \mathsf{res}) &\Longleftarrow \mathsf{take\text{-}max}_{\mathsf{L7}}(mb, \mathsf{res}) \\
\mathsf{take\text{-}max}_{\mathsf{L7}}(mb, mb) &\Longleftarrow \top
\end{aligned}$$

The fifth and eighth CHC represent release of $mb/ma$. The sixth and ninth CHC represent the determination of the return value $\mathsf{res}$.

#### 3.3 Correctness of the CHC Representation

Now we formally state and prove the correctness of the CHC representation.

*Notations.* We use $\{\!|\cdots|\!\}$ (instead of $\{\cdots\}$) for the intensional description of a multiset. $A \oplus B$ (or more generally $\bigoplus_\lambda A_\lambda$) denotes the multiset sum (e.g. $\{\!|0,1|\!\} \oplus \{\!|1|\!\} = \{\!|0,1,1|\!\} \neq \{\!|0,1|\!\}$).

**Readout and Safe Readout.** We introduce a few judgments to formally describe how read out data from the heap.

First, the judgment '$\mathrm{readout}_{\mathbf{H}}(*a :: T \mid v;\, \mathcal{M})$' (the data at the address $a$ of type $T$ can be read out from the heap $\mathbf{H}$ as the value $v$, yielding the memory footprint $\mathcal{M}$) is defined as follows.[21] Here, a *memory footprint* $\mathcal{M}$ is a finite multiset of addresses, which is employed for monitoring the memory usage.

$$\frac{\mathbf{H}(a) = a' \quad \mathrm{readout}_{\mathbf{H}}(*a' :: T \mid v;\, \mathcal{M})}{\mathrm{readout}_{\mathbf{H}}(*a \colon \mathsf{own}\, T \mid \langle v\rangle;\, \mathcal{M} \oplus \{\!|a|\!\})} \qquad \frac{\mathrm{readout}_{\mathbf{H}}(*a :: T[\mu X.T/X] \mid v;\, \mathcal{M})}{\mathrm{readout}_{\mathbf{H}}(*a :: \mu X.T/X \mid v;\, \mathcal{M})}$$

$$\frac{\mathbf{H}(a) = n}{\mathrm{readout}_{\mathbf{H}}(*a :: \mathsf{int} \mid n;\, \{\!|a|\!\})} \qquad \mathrm{readout}_{\mathbf{H}}(*a :: \mathsf{unit} \mid ();\, \varnothing)$$

$$\frac{\mathbf{H}(a) = i \in [2] \quad \text{for any } k \in [(\#T_{1-i} - \#T_i)_{\ge 0}],\ \mathbf{H}(a{+}1{+}\#T_i{+}k) = 0 \quad \mathrm{readout}_{\mathbf{H}}(*(a{+}1) :: T_i \mid v;\, \mathcal{M})}{\mathrm{readout}_{\mathbf{H}}\big(*a :: T_0{+}T_1 \mid \mathsf{inj}_i\, v;\, \mathcal{M} \oplus \{\!|a|\!\} \oplus \{\!|a{+}1{+}\#T_i{+}k \mid k \in [(\#T_{1-i} - \#T_i)_{\ge 0}]|\!\}\big)}$$

$$(n)_{\ge 0} := \max\{n, 0\}$$

$$\frac{\mathrm{readout}_{\mathbf{H}}\big(*a :: T_0 \mid v_0;\, \mathcal{M}_0\big) \quad \mathrm{readout}_{\mathbf{H}}\big(*(a{+}\#T_0) :: T_1 \mid v_1;\, \mathcal{M}_1\big)}{\mathrm{readout}_{\mathbf{H}}\big(*a :: T_0 {\times} T_1 \mid (v_0, v_1);\, \mathcal{M}_0 \oplus \mathcal{M}_1\big)}$$

For example, '$\mathrm{readout}_{\{(100,7),(101,5)\}}(*100 :: \mathsf{int} \times \mathsf{int} \mid (7,5);\, \{\!|100,101|\!\})$' holds.

Next, '$\mathrm{readout}_{\mathbf{H}}(\mathbf{F} :: \mathbf{\Gamma} \mid \mathcal{F};\, \mathcal{M})$' (the data of the stack frame $\mathbf{F}$ respecting the variable context $\mathbf{\Gamma}$ can be read out from $\mathbf{H}$ as $\mathcal{F}$, yielding $\mathcal{M}$) is defined as follows. $\mathrm{dom}\, \mathbf{\Gamma}$ stands for $\{x \mid x{:}^{\mathbf{a}}\, T \in \mathbf{\Gamma}\}$.

$$\frac{\mathrm{dom}\, \mathbf{F} = \mathrm{dom}\, \mathbf{\Gamma} \quad \text{for any } x \colon \mathsf{own}\, T \in \mathbf{\Gamma},\ \mathrm{readout}_{\mathbf{H}}(*\mathbf{F}(x) :: T \mid v_x;\, \mathcal{M}_x)}{\mathrm{readout}_{\mathbf{H}}(\mathbf{F} :: \mathbf{\Gamma} \mid \{(x, \langle v_x\rangle) \mid x \in \mathrm{dom}\, \mathbf{F}\};\, \bigoplus_{x \in \mathrm{dom}\, \mathbf{F}} \mathcal{M}_x)}$$

[21] Here we can ignore mutable/immutable references, because we focus on what we call *simple* functions, as explained later.

Finally, '$\mathrm{safe}_{\mathbf{H}}(\mathbf{F} :: \mathbf{\Gamma} \mid \mathcal{F})$' (the data of $\mathbf{F}$ respecting $\mathbf{\Gamma}$ can be *safely* read out from $\mathbf{H}$ as $\mathcal{F}$) is defined as follows.

$$\frac{\mathrm{readout}_{\mathbf{H}}(\mathbf{F} :: \mathbf{\Gamma} \mid \mathcal{F};\, \mathcal{M}) \quad \mathcal{M}\ \text{has no duplicate items}}{\mathrm{safe}_{\mathbf{H}}(\mathbf{F} :: \mathbf{\Gamma} \mid \mathcal{F})}$$

Here, the 'no duplicate items' precondition checks the safety on the ownership.

**COS-based Model.** Now we introduce the *COS-based model* (COS stands for concrete operational semantics) $f_{\Pi}^{\mathrm{COS}}$ to formally describe the expected input-output relation. Here, for simplicity, $f$ is restricted to one that does not take lifetime parameters (we call such a function *simple*; the input/output types of a simple function cannot contain references). We define $f_{\Pi}^{\mathrm{COS}}$ as the predicate (on values of sorts $(\!|T_0|\!), \dots, (\!|T_{n-1}|\!), (\!|U|\!)$ if $f$'s input/output types are $T_0, \dots, T_{n-1}, U$) given by the following rule.

$$\frac{\begin{array}{c}\mathbf{C}_0 \to_{\Pi} \cdots \to_{\Pi} \mathbf{C}_N \quad \mathrm{final}_{\Pi}(\mathbf{C}_N) \quad \mathbf{C}_0 = [f, \mathsf{entry}]\, \mathbf{F} \mid \mathbf{H} \quad \mathbf{C}_N = [f, L]\, \mathbf{F}' \mid \mathbf{H}' \\ \mathrm{safe}_{\mathbf{H}}\big(\mathbf{F} :: \mathbf{\Gamma}_{\Pi,f,\mathsf{entry}} \bigm| \{(x_i, v_i) \,|\, i \in [n]\}\big) \quad \mathrm{safe}_{\mathbf{H}'}\big(\mathbf{F}' :: \mathbf{\Gamma}_{\Pi,f,L} \bigm| \{(y, w)\}\big)\end{array}}{f_{\Pi}^{\mathrm{COS}}(v_0, \dots, v_{n-1}, w)}$$

$\mathbf{\Gamma}_{\Pi,f,L}$: the variable context for the label $L$ of $f$ in the program $\Pi$

**Correctness Theorem.** Finally, the correctness (both soundness and completeness) of the CHC representation is simply stated as follows.

**Theorem 1 (Correctness of the CHC Representation).** *For any program $\Pi$ and simple function $f$ in $\Pi$, $f_{\Pi}^{\mathrm{COS}}$ is equivalent to $\mathbf{M}^{\mathrm{least}}_{(\!|\Pi|\!)}(f_{\mathsf{entry}})$.*

*Proof.* The details are presented in the full paper. We outline the proof below.

First, we introduce *abstract operational semantics*, where we get rid of heaps and directly represent each variable in the program simply as a value with *abstract variables*, which is strongly related to *prophecy variables* (see § 5). An abstract variable represents the undetermined value of a mutable reference at the end of borrow.

Next, we introduce *SLDC resolution* for CHC systems and find a *bisimulation* between abstract operational semantics and SLDC resolution, whereby we show that the *AOS-based model*, defined analogously to the COS-based model, is *equivalent* to the least model of the CHC representation. Moreover, we find a *bisimulation* between concrete and abstract operational semantics and prove that the COS-based model is *equivalent* to the AOS-based model.

Finally, combining the equivalences, we achieve the proof for the correctness of the CHC representation. □

Interestingly, as by-products of the proof, we have also shown the *soundness of the type system* in terms of preservation and progression, in both concrete and abstract operational semantics. Simplification and generalization of the proofs is left for future work.

#### 3.4 Advanced Examples

We give advanced examples of pointer-manipulating Rust programs and their CHC representations. For readability, we write programs in Rust (with ghost annotations) instead of COR. In addition, CHCs are written in an informal style like § 1, preferring equalities to pattern matching.

*Example 3.* Consider the following program, a variant of `just_rec` in § 1.1.

```
fn choose<'a>(ma: &'a mut i32, mb: &'a mut i32) -> &'a mut i32 {
  if rand() { drop ma; mb } else { drop mb; ma }
}
fn linger_dec<'a>(ma: &'a mut i32) -> bool {
  *ma -= 1; if rand() >= 0 { drop ma; return true; }
  let mut b = rand(); let old_b = b; intro 'b; let mb = &'b mut b;
  let r2 = linger_dec<'b>(choose<'b>(ma, mb)); now 'b;
  r2 && old_b >= b
}
```

Unlike `just_rec`, the function `linger_dec` can modify the local variable of an arbitrarily deep ancestor. Interestingly, each recursive call to `linger_dec` can introduce a new lifetime *'b*, which yields arbitrarily many layers of lifetimes.

Suppose we wish to verify that `linger_dec` never returns `false`. If we use, like $\mathit{JustRec}_+$ in § 1.1, a predicate taking the memory states $h, h'$ and the stack pointer $sp$, we have to discover the quantified invariant: $\forall\, i \leq sp.\, h[i] \geq h'[i]$. In contrast, our approach reduces this verification problem to the following CHCs:

$$\begin{aligned}
&\mathit{Choose}(\langle a, a_\circ\rangle, \langle b, b_\circ\rangle, r) \impliedby b_\circ = b \wedge r = \langle a, a_\circ\rangle\\
&\mathit{Choose}(\langle a, a_\circ\rangle, \langle b, b_\circ\rangle, r) \impliedby a_\circ = a \wedge r = \langle b, b_\circ\rangle\\
&\mathit{LingerDec}(\langle a, a_\circ\rangle, r) \impliedby a' = a - 1 \wedge a_\circ = a' \wedge r = \mathsf{true}\\
&\mathit{LingerDec}(\langle a, a_\circ\rangle, r) \impliedby a' = a - 1 \wedge \mathit{oldb} = b \wedge \mathit{Choose}(\langle a', a_\circ\rangle, \langle b, b_\circ\rangle, \mathit{mc})\\
&\qquad\qquad \wedge\, \mathit{LingerDec}(\mathit{mc}, r') \wedge r = (r' \mathbin{\&\&} \mathit{oldb} >= b_\circ)\\
&r = \mathsf{true} \impliedby \mathit{LingerDec}(\langle a, a_\circ\rangle, r).
\end{aligned}$$

This can be solved by many solvers since it has a very simple model:

$$\begin{aligned}
\mathit{Choose}(\langle a, a_\circ\rangle, \langle b, b_\circ\rangle, r) &:\iff (b_\circ = b \wedge r = \langle a, a_\circ\rangle) \vee (a_\circ = a \wedge r = \langle b, b_\circ\rangle)\\
\mathit{LingerDec}(\langle a, a_\circ\rangle, r) &:\iff r = \mathsf{true} \wedge a \geq a_\circ.
\end{aligned}$$

*Example 4.* Combined with *recursive data structures*, our method turns out to be more interesting. Let us consider the following Rust code:[22]

```
enum List { Cons(i32, Box<List>), Nil } use List::*;
fn take_some<'a>(mxs: &'a mut List) -> &'a mut i32 {
  match mxs {
    Cons(mx, mxs2) => if rand() { drop mxs2; mx }
                      else { drop mx; take_some<'a>(mxs2) }
    Nil => { take_some(mxs) }
```

[22] In COR, `List` can be expressed as $\mu X.\mathsf{int} \times \mathsf{own}\, X + \mathsf{unit}$.

```
    }
}
fn sum(xs: &List) -> i32 {
  match xs { Cons(x, xs2) => x + sum(xs2), Nil => 0 }
}
fn inc_some(mut xs: List) -> bool {
  let n = sum(&xs); intro 'a; let my = take_some<'a>(&'a mut xs);
  *my += 1; drop my; now 'a; let m = sum(&xs); m == n + 1
}
```

This is a program that manipulates singly linked integer lists, defined as a recursive data type. `take_some` takes a mutable reference to a list and returns a mutable reference to some element of the list. `sum` calculates the sum of the elements of a list. `inc_some` increments some element of a list via a mutable reference and checks that the sum of the elements of the list has increased by `1`.

Suppose we wish to verify that `inc_some` never returns `false`. Our method translates this verification problem into the following CHCs.[23]

$$\begin{aligned}
&\mathit{TakeSome}(\langle [x|xs'], xs_\circ\rangle, r) \impliedby xs_\circ = [x_\circ|xs'_\circ] \land xs'_\circ = xs' \land r = \langle x, x_\circ\rangle\\
&\mathit{TakeSome}(\langle [x|xs'], xs_\circ\rangle, r) \impliedby xs_\circ = [x_\circ|xs'_\circ] \land x_\circ = x \land \mathit{TakeSome}(\langle xs', xs'_\circ\rangle, r)\\
&\mathit{TakeSome}(\langle [], xs_\circ\rangle, r) \impliedby \mathit{TakeSome}(\langle [], xs_\circ\rangle, r)\\
&\mathit{Sum}(\langle [x|xs']\rangle, r) \impliedby \mathit{Sum}(\langle xs'\rangle, r') \land r = x + r'\\
&\mathit{Sum}(\langle []\rangle, r) \impliedby r = 0\\
&\mathit{IncSome}(xs, r) \impliedby \mathit{Sum}(\langle xs\rangle, n) \land \mathit{TakeSome}(\langle xs, xs_\circ\rangle, \langle y, y_\circ\rangle) \land y_\circ = y + 1\\
&\qquad\qquad \land \mathit{Sum}(\langle xs_\circ\rangle, m) \land r = (m == n{+}1).
\end{aligned}$$

A crucial technique used here is *subdivision of a mutable reference*, which is achieved with the constraint $xs_\circ = [x_\circ|xs'_\circ]$.

We can give this CHC system a very simple model, using an auxiliary function $\mathsf{sum}$ (satisfying $\mathsf{sum}([x|xs']) := x + \mathsf{sum}(xs'),\ \mathsf{sum}([]) := 0$):

$$\begin{aligned}
\mathit{TakeSome}(\langle xs, xs_\circ\rangle, \langle y, y_\circ\rangle) &:\iff y_\circ - y = \mathsf{sum}(xs_\circ) - \mathsf{sum}(xs)\\
\mathit{Sum}(\langle xs\rangle, r) &:\iff r = \mathsf{sum}(xs)\\
\mathit{IncSome}(xs, r) &:\iff r = \mathsf{true}.
\end{aligned}$$

Although the model relies on the function $\mathsf{sum}$, the validity of the model can be checked without induction on $\mathsf{sum}$ (i.e. we can check the validity of each CHC just by properly unfolding the definition of $\mathsf{sum}$ a few times).

The example can be *fully automatically and promptly* verified by our approach using HoIce [12,11] as the back-end CHC solver; see § 4.

### 3.5 Discussions

We discuss here how our idea can be extended and enhanced.

[23] $[x|xs]$ is the cons made of the head $x$ and the tail $xs$. $[]$ is the nil. In our formal logic, they are expressed as $\mathsf{inj}_0(x, \langle xs\rangle)$ and $\mathsf{inj}_1()$.

*Applying Various Verification Techniques.* Our idea can also be expressed as a translation of a pointer-manipulating Rust program into a program of a *stateless functional programming language*, which allows us to use *various verification techniques* not limited to CHCs. Access to future information can be modeled using *non-determinism*. To express the value $a_\circ$ coming at the end of mutable borrow in CHCs, we just *randomly guess* the value with non-determinism. At the time we actually release a mutable reference, we just *check* `a' = a` and cut off execution branches that do not pass the check.

For example, `take_max`/`inc_max` in § 1.2/Example 1 can be translated into the following OCaml program.

```
let rec assume b = if b then () else assume b
let take_max (a, a') (b, b') =
  if a >= b then (assume (b' = b); (a, a'))
            else (assume (a' = a); (b, b'))
let inc_max a b =
  let a' = Random.int(0) in let b' = Random.int(0) in
  let (c, c') = take_max (a, a') (b, b') in
  assume (c' = c + 1); not (a' = b')
let main a b = assert (inc_max a b)
```

'`let a' = Random.int(0)`' expresses a *random guess* and '`assume (a' = a)`' expresses a *check*. The original problem "Does `inc_max` never return `false`?" is reduced to the problem "Does `main` never fail at assertion?" on the OCaml program.[24]

This representation allows us to use various verification techniques, including model checking (higher-order, temporal, bounded, etc.), semi-automated verification (e.g. on Boogie [48]) and verification on proof assistants (e.g. Coq [15]). The property to be verified can be not only partial correctness, but also total correctness and liveness. Further investigation is left for future work.

*Verifying Higher-order Programs.* We have to care about the following points in modeling closures: **(i)** A closure that encloses mutable references can be encoded as a pair of the main function and the 'drop function' called when the closure is released; **(ii)** A closure that updates enclosed data can be encoded as a function that returns, with the main return value, the updated version of the closure; **(iii)** A closure that updates external data through enclosed mutable references can also be modeled by combination of (i) and (ii). Further investigation on verification of higher-order Rust programs is left for future work.

*Libraries with Unsafe Code.* Our translation does not use lifetime information; the correctness of our method is guaranteed by the nature of borrow. Whereas

[24] MoCHi [39], a higher-order model checker for OCaml, successfully verified the safety property for the OCaml representation above. It also successfully and instantly verified a similar representation of `choose`/`linger_dec` at Example 3.

lifetimes are used for *static check* of the borrow discipline, many libraries in Rust (e.g. `RefCell`) provide a mechanism for *dynamic ownership check*.

We believe that such libraries with *unsafe code* can be verified for our method by a separation logic such as Iris [35,33], as RustBelt [32] does. A good news is that Iris has recently incorporated *prophecy variables* [34], which seems to fit well with our approach. This is an interesting topic for future work.

After the libraries are verified, we can turn to our method. For an easy example, `Vec` [58] can be represented simply as a functional array; a mutable/immutable slice `&mut[T]`/`&[T]` can be represented as an array of mutable/immutable references. For another example, to deal with `RefCell` [56], we pass around an *array* that maps a `RefCell<T>` address to data of type `T` equipped with an ownership counter; `RefCell` itself is modeled simply as an address.[25][26] Importantly, *at the very time we take a mutable reference* $\langle a, a_\circ \rangle$ *from a ref-cell, the data at the array should be updated into* $a_\circ$. Using methods such as pointer analysis [61], we can possibly shrink the array.

Still, our method does not go quite well with *memory leaks* [52] caused for example by combination of `RefCell` and `Rc` [57], because they obfuscate the ownership release of mutable references. We think that use of `Rc` etc. should rather be restricted for smooth verification. Further investigation is needed.

### 4 Implementation and Evaluation

We report on the implementation of our verification tool and the preliminary experiments conducted with small benchmarks to confirm the effectiveness of our approach.

#### 4.1 Implementation of RustHorn

We implemented a prototype verification tool *RustHorn* (available at `https://github.com/hopv/rust-horn`) based on the ideas described above. The tool supports basic features of Rust supported in COR, including recursions and recursive types especially.

The implementation translates the MIR (Mid-level Intermediate Representation) [45,51] of a Rust program into CHCs quite straightforwardly.[27] Thanks to the nature of the translation, RustHorn can just rely on Rust's borrow check and forget about lifetimes. For efficiency, the predicate variables are constructed by the granularity of the vertices in the control-flow graph in MIR, unlike the per-label construction of § 3.2. Also, assertions in functions are taken into account unlike the formalization in § 3.2.

[25] To borrow a mutable/immutable reference from `RefCell`, we check and update the counter and take out the data from the array.

[26] In Rust, we can use `RefCell` to naturally encode data types with circular references (e.g. doubly-linked lists).

[27] In order to use the MIR, RustHorn's implementation depends on the unstable nightly version of the Rust compiler, which causes a slight portability issue.

#### 4.2 Benchmarks and Experiments

To measure the performance of RustHorn and the existing CHC-based verifier SeaHorn [23], we conducted preliminary experiments with benchmarks listed in Table 1. Each benchmark program is designed so that the Rust and C versions match. Each benchmark instance consists of either one program or a pair of safe and unsafe programs that are very similar to each other. The benchmarks and experimental results are accessible at `https://github.com/hopv/rust-horn`.

The benchmarks in the groups `simple` and `bmc` were taken from SeaHorn (`https://github.com/seahorn/seahorn/tree/master/test`), with the Rust versions written by us. They have been chosen based on the following criteria: they (i) consist of only features supported by core Rust, (ii) follow Rust's ownership discipline, and (iii) are small enough to be amenable for manual translation from C to Rust.

The remaining six benchmark groups are built by us and consist of programs featuring mutable references. The groups `inc-max`, `just-rec` and `linger-dec` are based on the examples that have appeared in § 1 and § 3.4. The group `swap-dec` consists of programs that perform repeated involved updates via mutable references to mutable references. The groups `lists` and `trees` feature destructive updates on recursive data structures (lists and trees) via mutable references, with one interesting program of it explained in § 3.4.

We conducted experiments on a commodity laptop (2.6GHz Intel Core i7 MacBook Pro with 16GB RAM). First we translated each benchmark program by RustHorn and SeaHorn (version 0.1.0-rc3) [23] translate into CHCs in the SMT-LIB 2 format. Both RustHorn and SeaHorn generated CHCs sufficiently fast (about 0.1 second for each program). After that, we measured the time of CHC solving by Spacer [40] in Z3 (version 4.8.7) [69] and HoIce (version 1.8.1) [12,11] for the generated CHCs. SeaHorn's outputs were not accepted by HoIce, especially because SeaHorn generates CHCs with arrays. We also made modified versions for some of SeaHorn's CHC outputs, adding constraints on address freshness, to improve accuracy of representations and reduce false alarms.[28]

#### 4.3 Experimental Results

Table 1 shows the results of the experiments.

Interestingly, the combination of RustHorn and HoIce succeeded in verifying many programs with recursive data types (`lists` and `trees`), although it failed at difficult programs.[29] HoIce, unlike Spacer, can find models defined with primitive recursive functions for recursive data types.[30]

[28] For `base/3` and `repeat/3` of `inc-max`, the address-taking parts were already removed, probably by inaccurate pointer analysis.

[29] For example, `inc-some/2` takes two mutable references in a list and increments on them; `inc-all-t` destructively increments all elements in a tree.

[30] We used the latest version of HoIce, whose algorithm for recursive types is presented in the full paper of [11].

| | | | RustHorn | | SeaHorn w/Spacer | |
|---|---|---|---|---|---|---|
| *Group* | *Instance* | *Property* | *w/Spacer* | *w/HoIce* | *as is* | *modified* |
| simple | 01 | safe | <0.1 | <0.1 | <0.1 | |
| | 04-recursive | safe | 0.5 | timeout | 0.8 | |
| | 05-recursive | unsafe | <0.1 | <0.1 | <0.1 | |
| | 06-loop | safe | timeout | 0.1 | timeout | |
| | hhk2008 | safe | timeout | 40.5 | <0.1 | |
| | unique-scalar | unsafe | <0.1 | <0.1 | <0.1 | |
| bmc | 1 | safe | 0.2 | <0.1 | <0.1 | |
| | | unsafe | 0.2 | <0.1 | <0.1 | |
| | 2 | safe | timeout | 0.1 | <0.1 | |
| | | unsafe | <0.1 | <0.1 | <0.1 | |
| | 3 | safe | <0.1 | <0.1 | <0.1 | |
| | | unsafe | <0.1 | <0.1 | <0.1 | |
| | diamond-1 | safe | 0.1 | <0.1 | <0.1 | |
| | | unsafe | <0.1 | <0.1 | <0.1 | |
| | diamond-2 | safe | 0.2 | <0.1 | <0.1 | |
| | | unsafe | <0.1 | <0.1 | <0.1 | |
| inc-max | base | safe | <0.1 | <0.1 | false alarm | <0.1 |
| | | unsafe | <0.1 | <0.1 | <0.1 | <0.1 |
| | base/3 | safe | <0.1 | <0.1 | false alarm | |
| | | unsafe | 0.1 | <0.1 | <0.1 | |
| | repeat | safe | 0.1 | timeout | false alarm | 0.1 |
| | | unsafe | <0.1 | 0.4 | <0.1 | <0.1 |
| | repeat/3 | safe | 0.2 | timeout | <0.1 | |
| | | unsafe | <0.1 | 1.3 | <0.1 | |
| swap-dec | base | safe | <0.1 | <0.1 | false alarm | <0.1 |
| | | unsafe | 0.1 | timeout | <0.1 | <0.1 |
| | base/3 | safe | 0.2 | timeout | false alarm | <0.1 |
| | | unsafe | 0.4 | 0.9 | <0.1 | 0.1 |
| | exact | safe | 0.1 | 0.5 | false alarm | timeout |
| | | unsafe | <0.1 | 26.0 | <0.1 | <0.1 |
| | exact/3 | safe | timeout | timeout | false alarm | false alarm |
| | | unsafe | <0.1 | 0.4 | <0.1 | <0.1 |
| just-rec | base | safe | <0.1 | <0.1 | <0.1 | |
| | | unsafe | <0.1 | 0.1 | <0.1 | |
| linger-dec | base | safe | <0.1 | <0.1 | false alarm | |
| | | unsafe | <0.1 | 0.1 | <0.1 | |
| | base/3 | safe | <0.1 | <0.1 | false alarm | |
| | | unsafe | <0.1 | 7.0 | <0.1 | |
| | exact | safe | <0.1 | <0.1 | false alarm | |
| | | unsafe | <0.1 | 0.2 | <0.1 | |
| | exact/3 | safe | <0.1 | <0.1 | false alarm | |
| | | unsafe | <0.1 | 0.6 | <0.1 | |
| lists | append | safe | tool error | <0.1 | false alarm | |
| | | unsafe | tool error | 0.2 | 0.1 | |
| | inc-all | safe | tool error | <0.1 | false alarm | |
| | | unsafe | tool error | 0.3 | <0.1 | |
| | inc-some | safe | tool error | <0.1 | false alarm | |
| | | unsafe | tool error | 0.3 | 0.1 | |
| | inc-some/2 | safe | tool error | timeout | false alarm | |
| | | unsafe | tool error | 0.3 | 0.4 | |
| trees | append-t | safe | tool error | <0.1 | timeout | |
| | | unsafe | tool error | 0.3 | 0.1 | |
| | inc-all-t | safe | tool error | timeout | timeout | |
| | | unsafe | tool error | 0.1 | <0.1 | |
| | inc-some-t | safe | tool error | timeout | timeout | |
| | | unsafe | tool error | 0.3 | 0.1 | |
| | inc-some/2-t | safe | tool error | timeout | false alarm | |
| | | unsafe | tool error | 0.4 | 0.1 | |

**Table 1.** Benchmarks and experimental results on RustHorn and SeaHorn, with Spacer/Z3 and HoIce. "timeout" denotes timeout of 180 seconds; "false alarm" means reporting 'unsafe' for a safe program; "tool error" is a tool error of Spacer, which currently does not deal with recursive types well.

False alarms of SeaHorn for the last six groups are mainly due to problematic approximation of SeaHorn for pointers and heap memories, as discussed in § 1.1. On the modified CHC outputs of SeaHorn, five false alarms were erased and four of them became successful. For the last four groups, unboundedly many memory cells can be allocated, which imposes a fundamental challenge for SeaHorn's array-based approach as discussed in § 1.1.[31] The combination of RustHorn and HoIce took a relatively long time or reported timeout for some programs, including unsafe ones, because HoIce is still an unstable tool compared to Spacer; in general, automated CHC solving can be rather unstable.

### 5 Related Work

*CHC-based Verification of Pointer-Manipulating Programs.* SeaHorn [23] is a representative existing tool for CHC-based verification of pointer-manipulating programs. It basically represents the heap memory as an array. Although some pointer analyses [24] are used to optimize the array representation of the heap, their approach suffers from the scalability problem discussed in § 1.1, as confirmed by the experiments in § 4. Still, their approach is quite effective as automated verification, given that many real-world pointer-manipulating programs do not follow Rust-style ownership.

Another approach is taken by JayHorn [37,36], which translates Java programs (possibly using object pointers) to CHCs. They represent store invariants using special predicates *pull* and *push*. Although this allows faster reasoning about the heap than the array-based approach, it can suffer from more false alarms. We conducted a small experiment for JayHorn (0.6-alpha) on some of the benchmarks of § 4.2; unexpectedly, JayHorn reported '`UNKNOWN`' (instead of '`SAFE`' or '`UNSAFE`') for even simple programs such as the programs of the instance `unique-scalar` in `simple` and the instance `basic` in `inc-max`.

*Verification for Rust.* Whereas we have presented the first CHC-based (fully automated) verification method specially designed for Rust-style ownership, there have been a number of studies on other types of verification for Rust.

RustBelt [32] aims to formally prove high-level safety properties for Rust libraries with unsafe internal implementation, using manual reasoning on the higher-order concurrent separation logic Iris [35,33] on the Coq Proof Assistant [15]. Although their framework is flexible, the automation of the reasoning on the framework is little discussed. The language design of our COR is affected by their formal calculus $\lambda_{\mathsf{Rust}}$.

Electrolysis [67] translates some subset of Rust into a purely functional programming language to manually verify functional correctness on Lean Theorem Prover [49]. Although it clears out pointers to get simple models like our approach, Electrolysis' applicable scope is quite limited, because it deals with mutable references by *simple static tracking of addresses based on lenses* [20], not

[31] We also tried on Spacer $\mathit{JustRec}_+$, the stack-pointer-based accurate representation of `just_rec` presented in § 1.1, but we got timeout of 180 seconds.

supporting even basic use cases such as dynamic selection of mutable references (e.g. `take_max` in § 1.2) [66], which our method can easily handle. Our approach covers *all* usages of pointers of the safe core of Rust as discussed in § 3.

Some serial studies [27,3,17] conduct (semi-)automated verification on Rust programs using Viper [50], a verification platform based on separation logic with fractional ownership. This approach can to some extent deal with unsafe code [27] and type traits [17]. Astrauskas et al. [3] conduct semi-automated verification (manually providing pre/post-conditions and loop invariants) on many realistic examples. Because Viper is based on *fractional ownership*, however, their platforms have to use *concrete indexing on the memory* for programs like `take_max`/`inc_max`. In contrast, our idea leverages *borrow-based ownership*, and it can be applied also to semi-automated verification as suggested in § 3.5.

Some researches [65,4,44] employ bounded model checking on Rust programs, especially with unsafe code. Our method can be applied to bounded model checking as discussed in § 3.5.

*Verification using Ownership.* Ownership has been applied to a wide range of verification. It has been used for detecting race conditions on concurrent programs [8,64] and analyzing the safety of memory allocation [63]. Separation logic based on ownership is also studied well [7,50,35]. Some verification platforms [14,5,21] support simple ownership. However, most prior studies on ownership-based verification are based on fractional or counting ownership. Verification under *borrow-based ownership* like Rust was little studied before our work.

*Prophecy Variables.* Our idea of taking a future value to represent a mutable reference is linked to the notion of *prophecy variables* [1,68,34]. Jung et al. [34] propose a new Hoare-style logic with prophecy variables. In their logic, prophecy variables are not copyable, which is analogous to uncopyability of mutable references in Rust. This logic can probably be used for generalizing our idea as suggested in § 3.5.

### 6 Conclusion

We have proposed a novel method for CHC-based program verification, which represents a mutable reference as a pair of values, the current value and the future value at the time of release. We have formalized the method for a core language of Rust and proved its correctness. We have implemented a prototype verification tool for a subset of Rust and confirmed the effectiveness of our approach. We believe that this study establishes the foundation of verification leveraging borrow-based ownership.

**Acknowledgments.** This work was supported by JSPS KAKENHI Grant Number JP15H05706 and JP16K16004. We are grateful to the anonymous reviewers for insightful comments.
